# Locoregional Therapy for Intrahepatic Cholangiocarcinoma: The Role of Intra-Arterial Therapies

**DOI:** 10.3390/cancers15194727

**Published:** 2023-09-26

**Authors:** Leva Gorji, Hussein Aoun, Jeffrey Critchfield, Najeeb Al Hallak, Eliza W. Beal

**Affiliations:** 1 Department of Surgery, Kettering Health, Dayton, OH 45402, USA; leva.gorji@ketteringhealth.org; 2 Department of Interventional Radiology, Karmanos Cancer Institute, Wayne State University, Detroit, MI 48201, USA; aounh@karmanos.org (H.A.); critchfj@karmanos.org (J.C.); 3 Department of Oncology, Karmanos Cancer Institute, Wayne State University, Detroit, MI 48201, USA; alhallakm@karmanos.org; 4 Department of Surgery, Karmanos Cancer Institute, Wayne State University, Detroit, MI 48201, USA

**Keywords:** intrahepatic cholangiocarcinoma, transarterial embolization, transarterial chemoembolization, selective internal radiation therapy, Yttrium-90, radioembolization

## Abstract

**Simple Summary:**

The incidence of intrahepatic cholangiocarcinoma continues to rise, but survival remains dismal. Because malignancy often remains clinically indolent, treatment of the neoplasm becomes challenging. In advanced disease, locoregional therapies may be employed as a means of reducing toxicity and gaining disease control. A thorough understanding of these locoregional therapies will allow for optimal, individualized treatment. The intent of this review is to describethe role of intra-arterial therapies in the management of intrahepatic cholangiocarcinoma.

**Abstract:**

Intrahepatic cholangiocarcinoma (ICC) is a rare disease with a rising incidence. While surgical resection is the only curative option, the disease process is often identified in advanced stages, as this malignancy often remains clinically silent in early development. Only one-third of patients are eligible for resection at the time of diagnosis. For patients who cannot undergo resection, intra-arterial therapies are reasonable palliative treatment options; in rare occasions, these may be bridging therapies, as well. The premise of bland embolization and most chemoembolization intra-arterial therapies is that the arterial supply of the tumor is occluded to induce tumor necrosis, while radioembolization utilizes the arterial flow of the tumor to deliver radiation therapy. In this review, we discuss the use of transarterial embolization, transarterial chemoembolization, and selective internal radiation therapy for the treatment of ICC. Phase III randomized controlled clinical trials are difficult to tailor to this extremely rare and aggressive disease, but ultimately, further investigation should be pursued to define the patient population that will derive the greatest benefit from each modality.

## 1. Introduction

**Figure 1:**
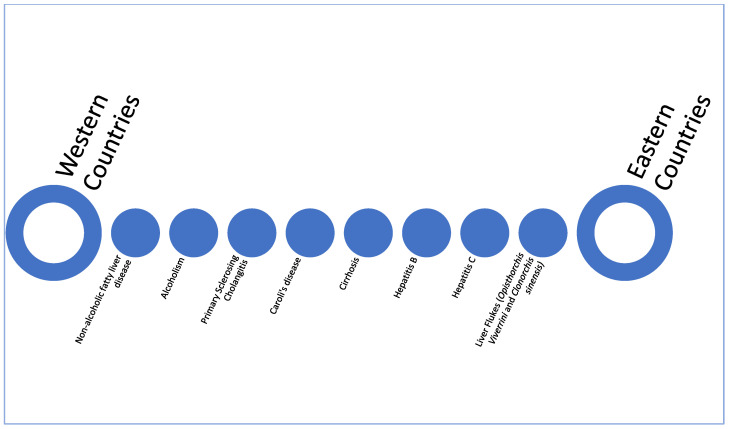
Risk factors associated with ICC according to the most common geographic distribution.

Cholangiocarcinoma (CCA) is an aggressive and rare heterogeneous group of cancers arising from the biliary tract. CCA is further subclassified as intrahepatic CCA (ICC) when arising from the segmental ducts or bile ductules, perihilar CCA (PCC) when emerging from the common hepatic duct or its main left and right branches, and distal CCA (DCC) when developing from the common bile duct [1,2,3]. Each subtype is associated with different clinical presentations, genomic alterations, and treatments [4]. Although the incidence of CCA is geographically variable, studies have demonstrated a rise in newly diagnosed cases globally [5,6,7]. ICC comprises nearly 10–15% of all primary hepatic malignancies, with the greatest incidence between the fifth to seventh decade of life [8]. Underlying geographic risk factors contribute to the variance in incidence (Figure 1) [9].

The malignancy is often clinically silent and presents in advanced stages with vague symptoms, including painless jaundice, weight loss, or cholangitis. ICC lesions may be classified as mass-forming, periductal infiltrating, intraductal, or mixed mass-forming and periductal. The purpose of the classification described by the Liver Cancer Study Group of Japan is for therapeutic or palliative morphology-based growth characteristics and treatment planning [10,11]. The American Joint Committee on Cancer (AJCC) provides a distinct staging system in their 8th edition to predict the prognosis of ICC. The AJCC staging system takes into account tumor size, vascular involvement, number of tumors, invasion of surrounding structures, nodal involvement, and metastatic disease (Table 1) [12]. Diagnosis and exclusion of metastasis are made with multiphasic thin slice CT or high-resolution MRI, endoscopic ultrasound (EUS)/endoscopic retrograde cholangiopancreatography (ERCP), and a CT of the chest with and without contrast. Baseline tumor markers, including carcinoembryonic antigen (CEA) and carbohydrate antigen 19-9 (CA 19-9) are obtained and may be trended for the progression of disease or response to treatment.

Surgical resection or transplantation remains the only curative therapy available for ICC [9]. Staging laparoscopy is recommended in some cases that appear resectable with significantly elevated CA19-9 to rule out occult peritoneal and omental metastases [13,14]. Neoadjuvant therapy has been described to downstage borderline and advanced tumors [15]. Adjuvant chemotherapy is often utilized as it shows survival benefits [16].

Although systemic therapy is imperative for the appropriate treatment of ICC, the optimal regimen continues to evolve and be defined. In the adjuvant setting, the PRODIGE-12 trial demonstrated no benefit in the use of gemcitabine and oxaliplatin for 12 cycles in comparison to surveillance following R0 or R1 resection of ICC [17]. In the BILCAP trial, patients following resection of their ICC or gallbladder malignancy were randomly assigned to oral capecitabine or observation. While the primary endpoint of OS did not reach statistical significance (*p* = 0.097), the median was 51.1 months in the capecitabine with 36.4 months in the observation group [18]. Therefore, the preferred adjuvant regimen is capecitabine [19].

The National Comprehensive Cancer Network (NCCN) does not have a preferred neoadjuvant regimen for ICC; however, it does advise multiple combinations of FOLFOX, gemcitabine, capecitabine, oxaliplatin, and cisplatin. The agents that are typically utilized concurrently with radiation include 5-fluorouracil and capecitabine [19].

There continues to be a shift toward the emphasis on precision medicine in ICC where the tumor microenvironment is accounted for in disease treatment as well. The tumor microenvironment is a complex interplay of cancer cells and the endogenous stroma impacting the progression of the malignancy [20,21]. The emerging role of targeted therapies that specifically aim to address the tumor microenvironment continues to be investigated. Monoclonal antibodies specifically bind to cancer cells interrupting their function and causing cytotoxicity, while small molecular inhibitors impair cellular function by interfering with intracellular signaling [22]. For patients with advanced BTC, the TOPAZ-1 trial combined immunotherapy with chemotherapy. Patients with BTC received durvalumab in combination with gemcitabine and cisplatin. The triple combination demonstrated an increased overall survival (12.8 vs. 11.5 months *p* = 0.021) in comparison to gemcitabine and cisplatin alone [23]. This regimen is now recommended as first-line systemic therapy for patients with advanced disease. The KEYNOTE-158 and KEYNOTE-028 trials administered 200 mg of pembrolizumab or 10 mg/kg of pembrolizumab in patients that demonstrated disease progression after surgical resection and determined a 6–18% antitumor activity irrespective of programmed death-ligand 1 expression [24]. Defining the role of systemic therapies is prudent and continues to be explored.

Locoregional treatment options for intrahepatic cholangiocarcinoma include microwave ablation (MWA), cryoablation, radiofrequency ablation (RFA), external beam radiation therapy (EBRT), transarterial embolization (TAE), transarterial chemoembolization (TACE), and selective internal radiation therapy (SIRT) with Yttrium-90 (Y-90) radioembolization [25,26]. These alternative-to-surgery treatment options can be used in patients who are not candidates for surgical resection or liver transplant, either due to their medical comorbidities or the location of the tumor in the liver.

**Figure 2:**
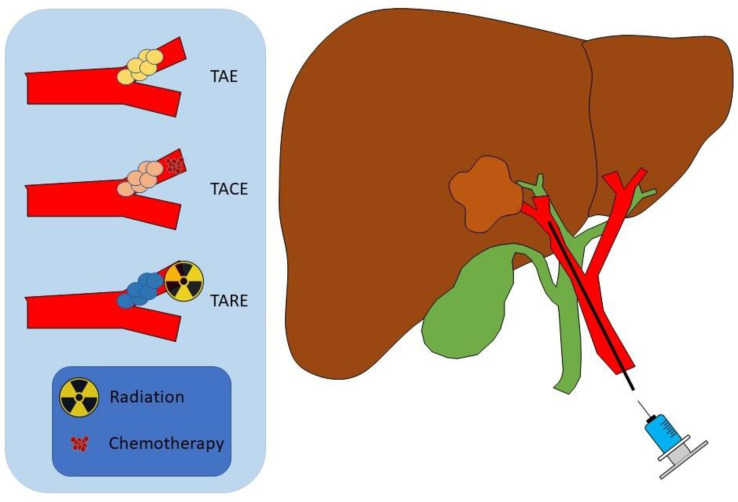
Schematic demonstrating transarterial embolization (TAE), transarterial chemoembolization (TACE), and transarterial radioembolization (TARE).

TAE, TACE, and SIRT are arterially based therapies (Figure 2). TAE, also known as ‘bland embolization’, is the injection of particles typically ranging from 40 to 900 microns into the arterial supply of a tumor to induce ischemia [27]. TACE includes the injection of antineoplastic drugs and iodized oil, which is then followed by the injection of gelatin sponge particles into the arterial supply of the tumor to reduce the nutrients and oxygen supply to the tumor [28]. While the use of radiation in ICC is controversial due to concerns about radioresistance, some studies have utilized Y-90 radioembolization as a therapeutic strategy in unresectable disease; this approach delivers a high dose of internal radiation to the malignancy through the hepatic artery [29]. In this review, we discuss the role of transarterial therapy and Y-90 radioembolization in treating ICC.

## 2. Transarterial Embolization

TAE has been utilized for the treatment of primary and secondary hepatic malignancies since its establishment in the 1980s. Microspheres or polyvinyl alcohol as embolic agents are injected into the main hepatic artery or hepatic artery segmental branches of the target tumor [30]. Studies suggest that there is not a significant difference in outcome when comparing microspheres and polyvinyl as embolization agents [31]. The premise of TAE, or bland embolization, is to deprive the malignancy of its blood supply and subsequently induce necrosis of the tumor. Successful TAE is demonstrated by the stasis of arterial flow supplying the tumor and completion of CT demonstrating contrast retention within the tumor [32,33]. The procedure is typically performed under intravenous sedation and begins with catheterization of a femoral or radial artery. An arteriogram of the superior mesenteric artery and celiac trunk is first performed to visualize any potential aberrant vasculature, followed by selective catheterization of the hepatic artery. Once the catheter is positioned in the vessel feeding the tumor, the small particles are injected to occlude the arterial supply to the tumor and the immediately surrounding normal tissue [31]. While many reports have demonstrated the utility of TAE in hepatocellular carcinoma (HCC), limited data are available on TAE in the setting of ICC [34]. In a multicenter study conducted by Hyder et al. of 198 patients with a median patient age of 61 years and a median tumor size of 8.1 cm, there was no significant survival difference on the basis of the type of IAT. Thirteen patients underwent TAE and demonstrated median OS of 14.3 months in patients with unresectable ICC compared to 13.4 months with conventional transarterial embolization (TACE), 10.5 months with drug-eluting bead (DEB), and 11.3 months with Y90 radioembolization (*p* = 0.46) [35]. In a six-patient study by Niu et al., with one of whom possessed ICC, TAE was associated with a partial response (PR) at 1-month post-procedure but demonstrated progressive disease at 3 months post-procedure by RECIST 1.1 criteria [36].

A broad range of adverse events with varying severities may occur following treatment; however, studies specific to the adverse events (AEs) of TAE for the treatment of ICC are extremely rare. Extrapolating from the treatment of HCC with TAE, common adverse symptoms from the procedure include abdominal pain, nausea, vomiting, and low-grade fever [33]. Postembolization syndrome (PES) is characterized by fever, abdominal pain, and leukocytosis in the immediate hours after the embolization of hepatic tumors often lasting days following intervention. It is hypothesized that this entity results from the inflammatory reaction caused by tissue ischemia. Approximately 30% of patients who undergo embolization experience PES; however, this percentage may be increased based on the amount of tissue included and the level of embolic ischemia that is induced [37,38]. Additional potential adverse events include the unlikely risk of embolization particles becoming exposed to the arterial supply of non-target tissue. Compromising the arterial supply of normal tissue can lead to gastrointestinal ulceration, hepatic abscess development, pancreatitis, and/or septicemia [39,40].

When comparing bland embolization to TACE using a propensity score analysis in patients with HCC, no significant difference was identified in the rate of AEs. However, TACE was associated with a greater radiological response (*p* = 0.390) with no difference in terms of overall survival (*p* = 0.390) [33]. Due to the rarity of ICC, no studies comparing bland embolization to other intra-arterial modalities were identified.

## 3. Transarterial Chemoembolization

TACE targets malignancy by administering high doses of chemotherapeutic agents directly through the hepatic artery while blocking tumor-feeding arteries and increasing the bioavailability of the chemotherapeutic agent [4]. Typically, contrast-enhanced cross-sectional imaging with CT and/or MRI is performed prior to the procedure to ensure there are no anatomic limitations to the procedure. The process begins with cannulating the femoral vessel and catheterizing the superior mesenteric artery with the injection of contrast for evaluation of aberrant anatomy. Next, the catheter is directed to the hepatic artery, and the vessel feeding the tumor is identified. Additional non-target feeding vessels to the tumor are coil embolized [41]. Conventional TACE (cTACE) regimens include the administration of a viscous anticancer-in-oil emulsion followed by an additional embolic agent under the pretense that the ischemia will enhance the cytotoxic effects of the chemotherapeutic agents. An alternative technique includes an embolic drug-eluting bead containing a chemotherapeutic agent, which has been reported to decrease the systemic distribution of the agent and increase intratumor drug dwell time due to selective occlusion of tumor-feeding arteries [42,43,44]. This is referred to as drug-eluting bead (DEB)-TACE.

Typically, greater than 50% of the liver’s volume should not be chemo-embolized simultaneously due to an increased risk of liver failure. In the circumstance where the tumor occupies greater than 50% of the liver, two separate procedures should be pursued. TACE should be avoided in patients with ascites, encephalopathy, jaundice, or variceal bleeding, as these factors are indicative of decompensated cirrhosis and TACE in these patients may result in hepatic failure [45]. TACE may still be utilized on an individualized basis in the setting of portal vein thrombus if there is adequate hepatopetal flow [46].

Multiple authors have demonstrated the benefits of cTACE as well as DEB-TACE for patients with ICC. However, the exact role of TACE in the treatment algorithm is still being defined, with many authors demonstrating its role in unresectable disease and some authors demonstrating its value in the adjuvant setting (Table 2) [44,47,48,49,50,51,52,53,54,55,56,57]. Gusani et al. compared TACE with gemcitabine only (n = 18) vs. gemcitabine followed by cisplatin (n = 2) vs. gemcitabine followed by oxaliplatin (n = 4) vs. gemcitabine and cisplatin in combination (n = 14), and gemcitabine and cisplatin followed by oxaliplatin (n = 4), and determined gemcitabine-cisplatin combination lead to increased OS in comparison to gemcitabine-alone at 13.8 months vs. 6.3 months in patients with unresectable cholangiocarcinoma [49]. Kuhlmann et al. demonstrated a PFS of 3.9 months and a median OS of 11.7 months in patients with ICC who underwent treatment with irinotecan DEB-TACE (iDEB-TACE) [50]. Poggi et al. demonstrated a median OS of 40 months in patients who underwent treatment with oxaliplatin-eluting microspheres—TACE (OEM-TACE) 40. Vogl et al. sought to evaluate the effectiveness of TACE in unresectable cholangiocarcinoma using mitomycin C, gemcitabine, mitomycin C and gemcitabine, or mitomycin c, gemcitabine, and cisplatin and demonstrated a 1-, 2-, and 3-year OS rate of 52%, 29%, and 10%, respectively; in this study, each patient underwent a mean of seven sessions [54].

TACE has also been used adjuvantly after surgical resection for ICC (Table 2). In the postoperative setting, Shen et al. demonstrated a 1-, 3-, and 5-year OS of 69.8%, 37.7%, and 28.3% in a group of patients with ICC who had previously undergone surgical resection with curative intent vs. a 1-, 3-, and 5-year survival of 54.2%, 25.0%, and 20,8%, respectively [52]. However, when Cheng et al. sought to demonstrate the benefit of adjuvant TACE in a study of 223 patients with microvascular invasion (MVI), only a subset of patients with elevated CA19-9 and those without lymphadenopathy exhibited a survival benefit [58]. These studies continue to be imperative in defining the optimal patient population and tumor characteristics for the use of TACE.

The most frequently reported AEs included abdominal pain, nausea, and low-grade fever (Table 2). Frequently, studies described a transient mild abdominal pain often associated with nausea or low-grade fever lasting from hours to nearly ten days [47,48,49,50,51,52,54,58,59]. Typically, this discomfort was described as grade 1 or 2 AE, meaning that pharmacologic treatment was required without the need for corrective intervention. Although significantly less common, grade 3 AEs require greater attention due to their severity and may result in the need for some type of further intervention. Grade 3 AEs often included severe abdominal pain, respiratory distress from over-sedation, and thrombocytopenia [47,49,50,51]. Grade 4 AEs result in a life-threatening complication, which also requires intervention. Grade 4 AEs reported included myocardial infarction, abscess development with subsequent thrombocytopenia, sepsis, and biliary leakage [49,50].

While specific guidelines exist for the use of TACE in HCC, there are no specific guidelines established for the use of TACE in ICC [60]. Commonly cited contraindications to TACE include extensive tumor infiltration, evidence of extra-hepatic disease with evidence of large tumor burden, encephalopathy indicating liver decompensation, portal vein thrombosis, or hepatic failure [45]. As studies continue to demonstrate the outcomes of TACE in ICC, optimal patient selection may be established. However, multiple studies have demonstrated its value in unresectable and recurrent diseases, while its role in adjuvant therapy requires further investigation.

## 4. Yittrium-90 Radioembolization

SIRT is a method of delivering radiotherapy to the tumor through the hepatic artery using Y-90 radiolabeled microspheres in addition to embolizing tumor-supplying arteries. Resin or glass microspheres that contain Y-90 are directly administered into the hepatic arteries that supply the tumor. Y-90-loaded microspheres are preferentially entrapped in the tumor vasculature, where they exert their cytotoxic effects; this phenomenon allows high doses of radiation to be distributed to the tumor while maintaining admissible radiation doses to the surrounding, normal hepatic tissue [29,61]. Y-90 is a β-emitter that emits radiation with a mean energy of 0.94 MeV, a mean tissue penetration of 2.5 mm, and a maximum tissue penetration of 11 mm. Over 90% of the Y-90 microsphere radiation is delivered during the first 11 days following treatment due to the 64.2 h half-life of the drug. Notably, if one gigabecquerel (GBq) of Y-90 was uniformly distributed through 1 kg of tissue, this would provide an absorbed dose of approximately 50 Gy [61,62,63]. The procedure is similar to other transarterial approaches, where the femoral or radial artery is first cannulated, and the catheter is directed toward the SMA, at which point a digital subtraction angiogram is performed in order to identify any aberrant vasculature or portal vein thrombus. Once the hepatic artery and the subsequent feeding vessel are identified, some centers recommend coil embolization of all extrahepatic arteries originating in close proximity to the Y-90 microsphere release [61]. A precursor mapping angiogram is also performed, where 99mTc-macroaggregated albumin (Tc-MAA) is injected into the feeding vessel. A nuclear medicine scan typically taking up to one hour is then employed and is used to detect extrahepatic shunting to predict the amount of radiation that will be distributed to the surrounding tissue in the lungs and gastrointestinal tract in addition to provisional dosimetry [64]. If the arterial anatomy and the Tc-MAA distribution do not preclude the patient from intervention, Y-90 beads are then later released inside the blood vessel [61].

There are two different microspheres that may be used for Y-90 embolization—glass and resin. Glass microspheres, in comparison to resin microspheres, are typically smaller (25 ± 10 μm vs. 35 ± 10 μm), have a greater density (3.6 g/dL vs. 1.6 g/dL), have a larger mean radioactivity per microsphere (2500 Bq vs. 50 Bq) and therefore have a smaller number of microspheres per Gbq (1.2 million vs. 60 million), come in a greater range of activities (3, 5, 7, 10, 15, 20 Bq vs. 3 Bq), and have less embolic effects (mild vs. moderate) [39]. Microsphere-associated adverse effects have also been reported; when comparing resin vs. glass microspheres, gastrointestinal ulceration rates were 1.4% vs. 0.1%, cholecystitis rates were 5% vs. 1.9%, hepatic abnormalities were 22.2% vs. 6.9%, and rates of hepatic encephalopathy were 8% vs. 2.8%, respectively [65].

Multiple observational studies have been conducted to evaluate the impact of Y-90 radioembolization on unresectable ICC. Studies investigating Y-90 therapy have demonstrated a benefit in OS and the temporary prevention of further tumor progression (Table 3) [66,67,68,69,70,71,72,73,74,75,76,77]. Some specific patient factors that have demonstrated a survival benefit with the use of Y-90 therapy include the patient’s Eastern Cooperative Oncology Group (ECOG) performance status. Studies have demonstrated an inverse relationship between the patient’s ECOG score and the benefit provided by Y-90 therapy [71,78]. Negative factors for OS included tumor burden (TB) >50%, a neutrophil/lymphocyte (N/L) ratio ≥3, and radiologic evidence of tumor progression [68,79]. Filippi et al. obtained FDG-PET CTs 6 weeks following Y-90 therapy to define the effect of radioembolization on the tumor and demonstrated a partial response (PR) in 14 patients and stable disease (SD) in three patients in a cohort of 17 patients. Furthermore, patients with a change in total lesion glycolysis (ΔTLG) > 50% had a mean OS of 79.6 weeks compared to patients with a ΔTLG < 50% who demonstrated a mean OS of 43.1 weeks (*p* < 0.001) [80]. As further investigation is pursued in the use of Y-90 therapy for ICC, additional patient qualities and tumor characteristics may be defined to optimize treatment benefits.

AEs from Y-90 radioembolization range broadly in severity (Table 3). The most common AE is postradioembolization syndrome (PRS), which produces symptoms of fatigue, nausea, vomiting, abdominal pain, and cachexia of varying degrees in 10–70% of patients and may last weeks [67,72]. Radioembolization-induced liver disease (REILD) may develop up to 8 months after intervention because of the hepatic necrosis caused by the radiation in up to 5% of patients. However, REILD prevention is ultimately based on optimal patient selection; patients with advanced liver disease, a baseline elevation in bilirubin, and an advanced Child–Pugh score may be at higher risk for developing REILD [76]. However, the risk of REILD was significantly reduced via a personalized dosimetry approach for patients with HCC undergoing Y-90 radioembolization with glass microspheres. The Dosisphere-01 trial demonstrated lower AEs and a greater objective tumor response when using personalized dosimetry (≥205 Gy targeted to the index lesion) in comparison to standard dosimetry (120 ± 20 Gy targeted to the perfused lobe) in patients with HCC [84]. Furthermore, personalized dosimetry software such as Simplicit90YTM (https://www.bostonscientific.com/en-US/products/cancer-therapies/simplicit90y-personalized-dosimetry-software.html) and MIM SurePlan (https://www.mimsoftware.com/nuclear_medicine/sureplan_mrt) are used for personalized dosimetry, allowing for the optimization of the radiation dose delivered to the tumor while minimizing the radiation to the surrounding tissue [85,86].

In an 81-patient study by Bargellini et al. evaluating the efficacy of Y-90 therapy in unresectable, 14.8% of patients reported symptoms of low-grade fever, abdominal pain, nausea, and vomiting lasting a maximum of seven days with no major AE [68]. Similar to TACE, the most common AEs of Y-90 therapy include grade 1 or 2 abdominal pain, nausea, vomiting, fatigue, or low-grade fever [66,67,68,69,70,71,72,78,80,81,82,83]. Some of the grade 3 and 4 AE events described include REILD, acute hepatic failure, cholangitis, ascites, severe abdominal pain, perforated cholecystitis, and tumor lysis syndrome with decompensated liver failure [66,67,69,71,72]. Furthermore, the risk of mortality is minute but present, with some studies reporting mortality with intervention in approximately 1.5% of patients [82,87]. Similar to all interventions, the risk profile and quality of life must be weighed against the survival benefit the treatment provides. 

The role of Y-90 therapy continues to have a progressive role in ICC, with some authors suggesting combination chemotherapy and Y-90 radioembolization having the therapeutic role of first-line therapy [67]. Some authors have advocated for the use of systemic chemotherapy in conjunction with Y-90 therapy to downsize the tumor and maximize treatment efficacy increasing the potential for resection [67]. Although definitive indications have not yet been established, Y-90 radioembolization has thus far demonstrated effective outcomes in unresectable ICC. In a study comparing Y-90 therapy to DEB-Tace in unresectable HCC, Y-90 therapy conferred superior tumor control and survival outcomes (30.2 months vs. 15.6 months, *p* = 0.006) [88]. It may be beneficial to apply this to ICC in a well-powered study to evaluate the consistency in outcomes between these two intraarterial therapies. Another potentially beneficial point of investigation may be the application of Holium-166 (Ho-166) as a microsphere for radioembolization as an alternative to the Y-90 therapy. Although not well studied in cholangiocarcinoma it has been studied in liver metastases and HCC, with positive results [89,90,91]. While the indications and contraindications of HO-166 and Y-90 therapy are similar, some of the proposed advantages include quantitative analysis regarding Ho-166 following treatment, MRI-guided injection with 3D inspection, and visualization of distribution. The isotope allows the performance of the scout and treatment utilizing the same particle [89].

Contraindications to Y-90 therapy include compromised liver function, including cirrhosis, ECOG >2 indicating a poor functional status, hyperbilirubinemia based on the severity of the tumor burden, impaired gastric perfusion, the result of >30 Gy radiation to the lungs, hepatopulmonary shunting with radiotherapy, severe and unmanageable contrast allergy with anaphylaxis, and uncorrectable coagulopathy [92,93].

## 5. Conclusions

The incidence of ICC continues to increase globally and demonstrates a notable geographic disparity of prevalence in Eastern Asian countries in comparison to Western countries [94,95]. This poses a significant clinical challenge since more than 50% of patients demonstrate unresectable disease on presentation. Surgical resection is the only curative option; however, recurrence rates remain high, at nearly 70% in less than 2 years [96,97]. Trans-arterial therapies with Y-90 radioembolization and TACE have become increasingly common for locoregional disease control and palliation. No defined criteria have been established for the utilization of either treatment option, or no guidelines currently exist for the preference of one treatment over the other. In a multi-institutional study by Hyder et al. of 198 patients treated with IAT, including TAE, TACE, and Y-90 radioembolization, survival did not differ based on the type of IAT utilized [35]. In a retrospective, observational study by Akinwande et al., there was no significant difference between TACE and Y-90 radiotherapy in terms of toxicity and disease control in the treatment of unresectable ICC [98]. Proponents of TACE highlight the diversity in the chemotherapeutic agents that can be utilized for greater targeting of the tumor. Advocates of TAE emphasize the same efficacy without the additional chemotherapeutic or radiation-related toxicity. Preference to Y-90 is given to those who argue that the vascularity of ICC is not equivalent to that of other primary liver malignancies, and embolization alone does not confer the same isolated benefit. Furthermore, Y-90 radioembolization often only requires one treatment, while TAE and TACE require multiple embolization sessions.

Limitations of the transarterial therapies may also be related to vascular leakiness, and successful targeting may be subject to tumor permeability and retention [99]. Tortuous and variable vascular tumor distribution may also cause disorganized distribution of these intraarterial therapies [100]. Future directions of the field include the ability to identify and monitor the permeability of the therapy to optimize disease control and management of adverse effects. Ultimately, further studies should be pursued to identify the optimal patient population that would benefit from each therapy.

## Figures and Tables

**Table 1:** American Joint Committee on Cancer TNM staging in their 8th edition [12].

Primary Tumor (T)	Nodal Involvement	Metastasis
T1a: Solitary tumor ≤5 cm without vascular involvement. T1b: Solitary tumor >5 cm without vascular involvement.	N1: Regional lymph node metastasis.	M1: Distant metastases.
T2: Solitary tumor with intrahepatic vascular involvement; multiple tumors +/− vascular involvement.		
T3: Tumor invading the visceral peritoneum.		
T4: Tumor invading local extrahepatic structures.		

**Table 2:** Outcomes of TACE in ICC.

Author	Study Period	Location	Patient Population	Approach	Outcomes	Toxicities
Unresectable Disease						
Aliberti et al. [47]	2000–2016	Italy	Unresectable ICC	N = 127 (N = 109 DEBDOX, N = 18 LIFDOX)	PR 15%, PD 5%, SD 80%, median OS 13.2 mo in patients with unresectable ICC	Abdominal pain, fever, nausea, and transaminitis. No grade 4 adverse events observed.
Liu et al. [55]	2016–2020	China	Unresectable ICC	N = 39, DEB-TACE	Median OS 11 mo, PFS 8 mo	Nausea, vomiting, abdominal pain, transaminitis, fever, and fatigue. One grade 3 AE of hepatic abscess development. No grade 4 AEs.
Ge et al. [48]	2008–2015	China	Median age 55 (20–85), Recurrent ICC	N = 275, N = 183 TACE, N = 92 PMCT	5-year OS improved TACE vs. PMCT, 21.4% vs. 6.1% (*p* = 0.034)	-
Gusani et al. [49]	2001–2007	USA	Median age 59 (36–86); 88% w/central ICC, 12% w/peripheral ICC; 45% with extrahepatic dsx.	N = 42	Median OS gem-cisTACE median OS 13.8 mo vs. gem-alone TACE 6.3 mo, respectively	Hyperbilirubinemia, elevated creatinine, thrombocytopenia, hyperglycemia, hypertension, pulmonary edema, and pancreatitis. Five pts had grade 3 AEs and 2 pts had grade 4 AEs.
Hu et al. [56]	2015–2019	China	Unresectable or progressive ICC	N = 35, apatinib plus DEB-TACE group (n = 10), apatinib plus cTACE group (n = 12), apatinib group (n = 13)	Apatinib plus DEB-TACE group: PFS 17 mo; OS 19.3 mo, apatinib plus cTACE group: PFS 10.3 mo; OS 14 mo, apatinib group: PFS 4.5 mo; OS 6.5 mo	Nausea, vomiting, abdominal pain, fever, and transaminitis.
Kuhlmann et al. [50]	2002–2010	Germany	Unresectable ICC	N = 46 with ICC, 23 pts treated with iDEB-TACE, 9 pts with cTACE with mitomycin C, 14 pts with ChT	iDEB-TACE PFS 3.9 mo, median OS 11.7 mo	Abdominal pain (34%), nausea (26.8%), fever (4.4%), hypertension (5.9%), alopecia (2.9%), and urticaria (1.5%) occurred in the cTACE and iDEB-TACE groups. Nine pts had grade 3 or 4 AEs. One death occurred in a cirrhotic, Child–Pugh A pt.
Luo et al. [44]	2015–2016	China	Primary HCC, ICC (n = 37), or secondary liver metastases	N = 37, DEB-TACE	Mean OS was 376 days, CR 8.1%, and ORR 67.6%	Nausea, vomiting, bone marrow toxicity, and fever. Grading severity not reported.
Poggi et al. [51]	2006	Italy	15 pts (8 with CRC LM, 7 with ICC), treatment with GEMOX prior to TACE	N = 7 patients with unresectable ICC treated with OEM-TACE.	SD 53.3%, PR 13.3%, PD 33.3% at a median FU 34 (6–92) mo median OS of 40 mo	Abdominal pain, low-grade fever, and nausea occurred in 53.2% of pts. Cholecystitis was seen in 2 pts, rash in 1 pt, and pancreatitis in 1 pt. There were no grade 4 AEs or deaths.
Vogl et al. [54]	1999–2010	Germany	Unresectable ICC, median age of 60.4 (37–87), Child–Pugh A or B.	N = 155 underwent TACE—24 pts Mitomycin C, 8 with Gemcitabine only, 54 with Mitomycin C + Gemcitabine, 29 in the Mitomycin C + Gemcitabine + Cisplatin.	1-, 2-, 3-year OS 52%, 29%, and 10% with no significant survival difference between groups, 8.7% PR, 57.4% SD, 33.9% PD	Abdominal pain, nausea, and vomiting in 9.6% of pts. No grade 3 or 4 complications.
Adjuvant TACE						
Cheng et al. [58]	2002–2015	China	resectable ICC with MVI	N = 223, p-TACE	p-TACE for ICC with MVI demonstrated benefit for OS and TTR in subgroup of patients with elevated CA19-9 and those w/o lymphadenopathy; otherwise, no association between p-TACE and OS or DFS	-
Shen et al. [52]	2002–2003	China	Recurrent ICC	N = 125, 53 pts underwent p-TACE vs. 72 pts in the non-TACE group	Median FU 18 (3–96) mo, 1-,3-, 5- year OS was higher in the adjuvant TACE after surgical resection group vs. non-TACE group 69.8 vs. 54.2, 37.7 vs. 25.0, and 28.3 vs. 20.8 (*p* = 0.045), respectively	Abdominal pain (35.8%), nausea/vomiting (47.1%), and fever (11.3%).
Wang et al. [53]	2014–2017	China	Pts with ICC who underwent curative-intent resection for ICC	N = 335, 39 with p-TACE vs. 296 non-TACE group	Median OS p-TACE 63 mo vs. 18 mo w/o p-TACE (*p* = 0.041)	-
Zhou et al. [57]	2015–2018	China	Unresectable or recurrent ICC who underwent DEB-TACE	N = 88 (58 without surgical intervention, 30 adjuvant)	Median PFS and OS 3 mo and 9 mo, respectively.	Nausea, vomiting, abdominal pain, transaminitis, low-grade fever, and cerebral infarct.

Abbreviations: AEs—Adverse Events, ChT—systemic chemotherapy, CRC LM—colorectal cancer with metastasis, cTACE—conventional trans-arterial chemoembolization, DEBDOX—doxorubicin microsphere drug-eluting bead, DEB-TACE—drug-eluting bead transarterial chemoembolization, Dsx—disease, FU—follow-up, GEMOX—gemcitabine oxaloplatin, ICC—intrahepatic cholangiocarcinoma, iDEB-TACE—trans-arterial embolization with irinotecan drug-eluting bead, LIFDOX—polyethylene glycol drug-eluting beads, MO—months, MVI—microvascular invasion, ORR—objective response rate, OS—overall survival, PD—progressive disease, PFS—progression-free survival, PMCT—Percutaneous Microwave Coagulation Therapy, p-TACE—postoperative trans-arterial embolization, PTS—patients, PR—partial response, SD—stable disease, TP—tumor progression, USA—United States of America, W/o—without.

**Table 3:** Outcomes of Y-90 in unresectable ICC.

Author	Study Period	Location	Patient Population	Approach	Outcomes	Toxicities
Bargellini et al. [68]	2008–2017	Italy	Unresectable ICC	N = 81, 3 treatment groups (a: 35 chemotherapy-naïve pts, b: 19 pts with disease control after first-line chemo, c: 27 pts with disease progression after first-line chemo)	Median OS 14.5 mo did not differ significantly among the treatment groups. TB > 50%, N/L ratio ≥ 3, and radiologic progression independent, negative factors for OS (*p* < 0.05)	Abdominal pain, nausea, vomiting
Buettner et al. [66]	2006–2017	Netherlands, UK, USA	Unresectable ICC	N = 115, 92 pts treated with resin microspheres, 22 pts treated with glass microspheres, 1 treated with both	Median OS 29 mo, and 1-, 3-, and 5- year survival 85%, 31%, 8%	Fatigue, pain, nausea, vomiting, DVT, generalized weakness, gastrointestinal hemorrhage, REILD, neuropathy
Camacho et al. [81]	2009–2012	USA	Unresectable, chemorefractory ICC	N = 21, treatment with Y-90 resin microspheres	Median OS from Y-90 tx was 16.3 mo	-
Depalo et al. [77]	2013–2018	Italy	Unresectable ICC	N = 15	Median of tumor average absorbed dose was 93 Gy, median of α and α3D parameters was 0.005 Gy−1 and 0.007 Gy−1, respectively. Tumor volume and tumor absorbed dose were prognostic indicators of TTP	-
Edeline et al. [67]	2013–2016	France	Unresectable ICC, chemotherapy, and intra-arterial therapy naïve	N = 41, Y-90 therapy, Phase 2 clinical trial	Combination of chemotherapy (cis+gem) and RE median PFS 14 mo (8–17 mo) and median OS 22 mo (14–52 mo)	Abdominal pain (41%), nausea (49%), diarrhea (29%), constipation (17%), diarrhea (29%), dysphagia (5%), neutropenia (73%), thrombocytopenia (63%)
Filippi et al. [80]		Italy	Unresectable, chemorefractory ICC	N = 17, treatment with Y-90 glass or resin microspheres	FDG-PET CT was performed 6 weeks following Y-90 tx. Fourteen pts had a PR and 3 pts with SD. No pts demonstrated CR; Pts with ΔTLG > 50% and ΔTLG < 50% had a mean OS of 79.6 and 43.1 weeks, respectively (*p* < 0.001)	Abdominal pain (35.3%), moderate gastritis (11.7%), severe gastritis (5.8%)
Gangi et al. [71]	2009–2016	USA	Unresectable ICC	N = 85, treatment with Y-90 glass microspheres	Median OS 12 mo, increased with ECOG score < 2 compared to ECOG ≥ 2 (18.5 vs. 5.5 mo *p* = 0.0012), well-differentiated histology (18.6 vs. 9.7 mo *p* = 0.012), and solitary tumors vs. multifocal (25 vs. 6.1 mo *p* = 0.006)	Abdominal pain (18.8%), weight loss (7.1%), ascites (5.9%), biochemical toxicities (hyperbilirubinemia, transaminitis) (53%)
Gupta et al. [82]	2004–2020	USA	Unresectable ICC	N = 136, treated with Y-90 glass microspheres	Median OS 14.2 mo; At 3 mo, 24.4% had a PR, 74.4% had SD, and 1.2% had PD	Fatigue (72%), abdominal pain (31.1%), hypoalbuminemia (43.9%), elevated alkaline phosphatase (30.9%)
Hoffman et al. [78]	2007–2010	Germany	Unresectable ICC	N = 33, treatment with Y-90 resin microsphere	Median OS 22 mo posttreatment	Abdominal pain (84.8%), nausea (60.6%), vomiting (27.3%), hyperbilirubinemia (69.7%)
Levillain et al. [70]	2004–2018	Belgium	Unresectable, chemorefractory ICC	N = 58, 30 pts with previous curative-intent liver resection, 28 pts w/o previous resection treated with Y-90 resin microspheres	Median OS 10.3 mo, 1- and 2-year survival rates after Y-90 were 40% and 22%	-
Paprottka et al. [76]	-	Germany	Unresectable ICC	N = 73, treatment with Y-90 resin microspheres	Median PFS 6.4 mo OS 18.9 mo, respectively; Patients with a tumor burden ≤25% had a significantly longer OS (15.2 vs. 6.6 mo; *p* = 0.036); Median PFS longer for patients with multiple TARE cycles (24.4 vs. 5.8 mo; *p* = 0.04)	Nausea, vomiting, pain, fever, gastritis, pancreatitis
Paz-Fumagalli et al. [72]	2016–2020	USA	Unresectable ICC	N = 28, treatment with Y-90 glass microspheres	30 mo OS of 59% in patients with unresectable ICC; 6 patients were downsized to resection post-Y-90 therapy	Abdominal pain, fever, perforated cholecystitis
Rafi et al. [83]	2002–2010	USA	Unresectable, chemorefractory ICC	N = 19, treatment with Y-90 resin microspheres	Median OS from diagnosis and first Y90 tx was 752 [95% CI374–1130] and 345 (95% CI 95–595) days, respectively. Higher ECOG scores and extrahepatic metastasis were associated with worse outcomes	Fatigue (21%), abdominal pain (32%), thrombocytopenia (5%)
Riby et al. [75]	1997–2017	France	Resectable ICC and unresectable ICC (underwent neoadjuvant therapy for downstaging)	N = 169, 137 surgically resectable, 32 with downstaging intervention (13 with neoadjuvant chemotherapy, and 19 with Y-90)	Median OS not statistically significant; 32.3 mo in the primary surgery group, and 45.9 mo in the downstaging group (*p* = 0.54)	-
Sarwar et al. [74]	2015–2020	USA	Unresectable ICC	N = 31, treatment with Y-90 resin microspheres; Neoadjuvant use for patients with tumor proximity to middle hepatic vein or insufficient liver remnant in 21 patients	Median PFS 5.4 mo; Median OS 22 mo	Nausea, vomiting, abdominal pain, pneumonia, transaminitis; 9 patients experienced grade 3 events, and 1 patient experienced a grade 4 event (obstructive jaundice)
Schatka et al. [73]	2009–2016	Germany	Unresectable ICC with hepatic metastases; Additional nodal (19 pts), bone (2 pts), and lung (2 pts) metastases included	N = 39, treatment with Y-90 resin microspheres	Median OS 8 mo. ECOG ≥1 (HR 3.8), high ggt (HR 1.002), AST/ALT quotient (HR 1.86), high CA19-9 (HR 1.00), and dose reduction ≥40% (HR 3.8) were poor prognostic indicators of OS; Median OS 15.3 mo with 0 risk factors, 7.6 mo with 1 risk factor, and 1.8 months with 2 risk factors (*p* < 0.001)	Nausea, vomiting, fever, abdominal pain, angina
White et al. [69]	2013–2017	UK	Unresectable ICC	N = 61, treatment with Y-90 microspheres	Median OS was 8.7 mo (5.2–12.1 mo); PFS was 2.8 mo (2.6–3.1 mo)	Abdominal pain, fatigue, fever, diarrhea, tumor lysis syndrome, portal vein thrombosis, liver decompensation

Abbreviations: ALT—alanine aminotransferase, AST—aspartate aminotransferase, CA19-9—carbohydrate antigen 19-9, Cis—cisplatin, CR—complete response, DVT—deep vein thrombosis, ECOG—Eastern Cooperative Oncology Group, FDG-PET—fluorodeoxyglucose-positron emission tomography. Gem—gemcitabine, GY—gray, HR—hazard ratio, ICC—intrahepatic cholangiocarcinoma, N/L—neutrophil-to-lymph node, MO—months, OS—overall survival, PD—progressive disease, PFS—progression-free survival, PR—partial response, RE—radioembolization, REILD—radioembolization-induced liver disease, SD—stable disease, TARE—transarterial radioembolization, TB—tumor burden, TTP—time-to-progression, Tx—treatment, UK—United Kingdom, USA—United States of America, Y-90—yttrium-90, ΔTLG—change in total lesion glycolysis.

## Abbreviations

AEs—adverse events, AJCC—American Joint Committee on Cancer, ALT—alanine aminotransferase, AST—aspartate aminotransferase, CA 19-9—Carbohydrate Antigen 19-9, CCA—cholangiocarcinoma, CEUS—contrast-enhanced ultrasound, ChT—systemic chemotherapy, CI—contraindications, Cis—cisplatin, CRC LM—colorectal cancer with liver metastasis, cTACE—Conventional Transarterial Embolization, DCC—distal cholangiocarcinoma, DEB—drug-eluting bead, DEBDOX—doxorubicin microsphere drug-eluting beads, DEB-TACE—drug-eluting bead transarterial chemoembolization, DFS—disease-free survival, Dsx—disease, DVT—deep vein thrombosis, EBRT—external beam radiation therapy, EC—exclusion criteria, ECOG—Eastern Cooperative Oncology Group performance status, ERCP—endoscopic retrograde cholangiopancreatography, EUS—endoscopic ultrasound, FDG-PET—fluorodeoxyglucose-positron emission testing, Gbq—gigabecquerel, GEMOX—gemcitabine oxaliplatin, Gy—gray, HAI—hepatic artery infusion pump, HCC—hepatocellular carcinoma, Ho-166—Homium-166, HR—hazard ratio, IC—inclusion criteria, ICC—Intrahepatic Cholangiocarcinoma, iDEB-TACE—Irinotecan Drug-Eluting bead transarterial chemoembolization, LIFDOX—polyethylene glycol drug-eluting beads, MC—most common, Mo—months, MRCP—magnetic resonance cholangiopancreatography, MS—median survival, MVI—microvascular invasion, MWA—microwave ablation, NAFLD—non-alcoholic fatty liver disease, NCCN—National Comprehensive Cancer Network, N/L—neutrophil-to-lymph node, OEM-TACE—oxaliplatin-eluting microsphere transarterial chemoembolization, ORR—objective response rate, OS—overall survival, PCC—perihilar cholangiocarcinoma, PD—progressive disease, PEG—polyethylene glycol drug-eluting microsphere, PES—post-embolization syndrome, PH—partial hepatectomy, PFS—progression-free survival, PMCT—percutaneous microwave coagulation therapy, PP—patient population, p-TACE—postoperative trans-arterial embolization, TARE—transarterial radioembolization, PSC—primary sclerosis cholangitis, Pts—patients, PR—partial response, REILD—radioembolization-induced liver disease, RFA—radiofrequency ablation, SD—stable disease, SE—side effects, SIRT—selective internal radiation therapy, RR—recurrence rate, TACE—transarterial chemoembolization, TAE—transarterial embolization, TB—tumor burden, Tc-MAA—^99m^Tc-macroaggregated albumin, TP—tumor progression, TTP—time-to-progression, UK—United Kingdom, USA—United States of America, Y-90—Yttrium-90, ΔTLG—total lesion glycolysis.
